# Clinical and service integration. The route to improved outcomes

**Published:** 2012-10-12

**Authors:** Christin Juhnke

**Affiliations:** Institute Health Economics and Healthcare Management, Hochschule Neubrandenburg (Germany)

## Background

Integration can take a variety of forms at the macro-, meso- and micro-levels. On the macro-level systems aim to deliver integrated care on a high-level of performance to the population they serve. Meso-level systems focus on the integrated care needs of a particular group or population with same diseases or same conditions. Consequently, integrated systems on the micro-level seek to improve care co-ordination for individual patients and carers.

Based on experiences from the USA, UK, and Europe the report comprises successful integrated systems and summarizes relevant evidence about high-profile systems.

The book is technically divided into five parts. Following usual convention in the literature, the first and the last section include Introduction and Discussion/Conclusion. The three middle sections, however, are each concerned with one specific level of integration: macro-, meso- and micro-level. For reasons of clarity and comprehensibility this review follows this structure.

In Chapter I the authors provide a comprehensive overview of the meanings of different terms, diverse forms of integration in health care and their differentiation and interrelationships. Definitions include ‘fragmentation’, ‘integration/integrated care’ as well as its forms of organizational, functional, service, clinical, normative, systemic, horizontal, vertical, real, virtual/contractual and commissioner integration.

## Macro-level integration

In the report macro-level integration is described using examples from the US. These systems have high levels of organization and tend to perform better than the fragmented forms of care. Each of the described approaches can be categorized as either complete provider and commissioner integration or provider integration or integrated medical groups.

Based on a clear and reasonable research structure consisting of a detailed explanation and description of the integrated systems followed by an assessment of the system’s impact in the national health system based on evaluations, survey data and national ranking the following five integrated care programs are examined:

- **Kaiser Permanente:** Virtually integrated system in which provider remain distinct organizations that co-operate based on exclusive contracts.
- **Veterans Health Administration/VA:** Real integrated system of regionally based integrated service networks across all care settings.
- **Geisinger Health System:** Hospital system, partially integrated in terms of provision and commissioning.
- **Mayo Clinic:** Multispecialty groups practice consisting of hospitals and medical offices without own health plan.
- **Integrated Medical Groups (multispecialty medical groups):** Composition of primary, community physicians from diverse specialties that are either directly employed or have an exclusive contract or have the budget responsibility. Kaiser, VA and Mayo Clinic are seen as integrated medical groups.

Moreover, the individual systems are compared among themselves and to the NHS in the UK.

## Meso-level integration

Meso-level integration seeks to integrate care for particular groups of patients and population. These programs are mostly focused on older people and those suffering from specific conditions.

Following this dichotomy the authors present examples of integration for older people and condition-specific integration. As in the 2nd section of the report the structure of description and impact-analysis based on evaluations and performance measures is used to examine nine systems:

- **Program for all-inclusive care for the elderly:** US-based integrated provider model aimed at maintaining frail older people in the community.
- **System of integrated services for aged persons:** Canadian integrated provider model seeking to overcome fragmented health and social care for elderly. Ended in 2001.
- **PRISMA:** Canadian model of integration and co-ordination of services across provider network to ensure functional autonomy.
- **Rovereto:** Italian model aimed to integrate medical and social services in a continuum of care using case managers. Pilot in 1990s.
- **Vittorio Veneto:** Italian model aimed to improve the integration and co-ordination of older people’s health and social care. Pilot in 1990s.
- **Torbay:** UK-based model using integrated health and social care teams proactively managing the care of vulnerable elderly in cooperation with GPs.
- **Disease management programs:** Mostly multicomponent, consisting of a variety of different elements and aim to reduce deficits in the care for long-term conditions such as coronary health disease, COPD or diabetes.
- **Chains of care:** Swedish-based way of co-ordination and integration of care across organizations and professions. Focused on certain conditions and based on clear care pathways to improve quality of care.
- **Managed clinical networks:** Scottish system seeking to broker care across providers from all sectors that does not require the creation of new organizational entities.

The first six examples display ways of integration for older people. In addition to the detailed description the authors also provide a very clear table summarizing the key features and impacts of each system.

## Micro-level integration

Micro-level integration is focused on the co-ordination of care for individual patients and carers. Responsibility for care co-ordination is often assigned to a specific individual or a team of care providers trying to decrease fragmentation.

Six ways of micro-level integration have been identified by the authors:

- **Care planning and care coordinators:** Refers to the planning, coordinating, managing and reviewing an individual’s care. The process of care planning is basically done by a care coordinator.
- **Case management:** Assignment of a case manager/care team to each patient that is responsible for assessing needs, developing care plans, organization of care, monitoring the quality of care, and the maintaining of the contact with the patient/family.
- **Patient-centred medical homes and virtual wards:** US-based system based on an accountable individual/care team and shared information systems and focused on care delivery for individuals with special care needs.
- **Personal health budgets:** Shifting of autonomy over care to patients by giving them responsibility to administer own health budget. Also referred to as self-directed care or individual budget.
- **Use of information technology:** Facilitates the timely and efficient flow of information across provider.
- **Use of telecare and telehealth:** Enable integrated care especially for long-term conditions by focusing on reducing avoidable and costly use of healthcare resources.

The final, fifth chapter of the report takes a closer look at the situation in the UK as well as the implications resulting from the examples. The authors clearly state that action is needed at all three levels to ensure that integration and cooperation deliver better outcomes.

## Conclusion and recommendations

This report reviews ways of achieving closer integration for individual service users and carers through care coordination. Very positive: Each section closes with a summary of the key messages of the section. This provides a valuable overview to all readers, allowing them to get first useful information in a short period of time.

The reader has to be aware that the paper is primarily aimed at NHS personnel confronted with the re-organization of the UK-system. However, this report has a special value for all policy-makers faced with the question of overcoming fragmentation of care.

